# Examining the Representation of Minority Race and Ethnicity Groups in Breast Cancer Clinical Trials in the USA Published 2012–2022

**DOI:** 10.1245/s10434-026-19214-y

**Published:** 2026-02-23

**Authors:** Katrina Dimaano, Otilio Castillo, Ashkan Moazzez, Junko Ozao-Choy, Christine Dauphine

**Affiliations:** 1 Division of Surgical Oncology, Department of Surgery, Harbor-UCLA Medical Center, Torrance, CA USA; 2 School of Medicine, University of California, Irvine, Irvine, CA USA

## Abstract

**Background:**

Diversity of study populations in clinical trials is essential to addressing health outcome disparities in cancer care. The objective of this study was to examine the inclusion of minority race/ethnicity groups in breast cancer clinical trials published in the United States of America (USA) 2012–2022.

**Patients and Methods:**

Breast cancer clinical trials published as a full manuscript in the USA 2012–2022 were identified. Trial characteristics and race/ethnicity of subjects were collected to assess the degree of reporting and inclusion of minority populations. Participation to Prevalence Ratios (PPRs) were utilized as a measure of inclusion.

**Results:**

In 1068 trials (*n* = 871,683 subjects), race/ethnicity was reported in 785 (73.5%), increasing from 2012 to 2016 (68.8%) to 2017 to 2022 (79.3%, *p* < 0.001). Higher rates of race/ethnicity reporting were independently associated with later publication year, randomized or phase II trials, and those reporting social determinants of health. Of the 732,969 subjects for whom race/ethnicity was reported, 16,551 (2.3%) were Asian American/Pacific Islander (Asian), 43,288 (5.9%) were Black/African American, 22,464 (3.1%) were Hispanic/Latinx, and 570,897 (77.9%) were Non-Hispanic White. A PPR above 0.8 was achieved in 27.3% of trials that reported race for Asian subjects, 44.7% for Black/African American subjects, 27.4% for Hispanic/Latinx subjects, and in 5.7% for all three minority groups. All four primary race groups in the USA were represented in 24.7% of trials, and race/ethnicity was included as a factor in outcome analysis in 8.7%.

**Conclusions:**

Although race/ethnicity reporting has improved, minority populations remain gravely underrepresented in breast cancer clinical trials, limiting the generalizability of trial results in these populations.

The National Institutes of Health (NIH) defines a clinical trial as a research study in which human subjects are prospectively assigned to one or more interventions to evaluate the effects of those interventions on health-related biomedical or behavioral outcomes.^1^ These trials form the strongest evidentiary basis upon which practice guidelines and treatment algorithms are established. The generalizability of study results to minority populations is conditional to the racial diversity of the subjects included in clinical research.

Breast cancer is awarded the highest proportion of research funding grants annually (10.8%) and receives the highest overall funding (11.2%) globally compared with other primary cancers.^2^ As a possible reflection of this research effort, 5 year mortality for early invasive breast cancer decreased from 14.4 to 4.9% in an English cohort from 1993 to 2015.^3^ Despite this, the breast cancer mortality gap among Black and White women persists, with Black women having 42% higher mortality than White women.^4^ To address these health disparities, it is vital that research leading to advancements in breast cancer treatment and the concomitant improvement in outcome is inclusive of all populations affected.

To address underrepresentation of minority race/ethnicity groups in clinical trials, the National Institutes of Health (NIH) published its Revitalization Act of 1993, establishing guidelines for the inclusion of women and racial and ethnic minority populations in clinical research.^5^ The United States (USA) Department of Health and Human Services (DHHS) subsequently mandated in its Policy Statement on Inclusion of Race and Ethnicity in DHHS Data Collections Activities the collection and reporting of race/ethnicity in all research funded or sponsored by its agencies.^6^ In 2021, the American Medical Association (AMA) released guidelines aiming to further improve the equity, consistency, and transparency of race/ethnicity reporting in medical journals.^7^ The impact of these efforts on race/ethnicity reporting and representation of racial minorities in breast cancer clinical trials is not well-described. Therefore, the objective of this study was to examine the degree of inclusion of minority populations in the conduct, analysis, and reporting of breast cancer clinical trials in the USA published between 2012 and 2022.

## Patients and Methods

To identify breast cancer clinical trials for this analysis, the National Library of Medicine’s PubMed search engine was utilized.^8^ A search was performed in December 2022 for all clinical trials using the terms “breast cancer,” “breast carcinoma,” and “breast malignancy” for publication years 2012–2022 and subsequently filtered to include those involving human subjects, published in English, and for which a full manuscript was available. Electronic versions of each manuscript were obtained through the institutional libraries at Harbor-UCLA Medical Center and the University of California, Irvine. Trials were excluded if the research was performed outside of the USA, published in a nonpeer reviewed journal, or consisted of a meta-analysis or a secondary long-term follow up of a previously published trial.

The following data was extracted from the clinical trials which comprised the study population: number of total subjects, number of subjects according to race/ethnicity, collection of other social determinants of health, type of intervention studied, phase/type of clinical trial, and funding source. Whether AMA race-reporting guidelines^7^ were followed and whether race/ethnicity were included in trial outcome analysis were also collected. Race and ethnicity were recorded according to the specificity available in each individual manuscript and stratified as: American Indian/Alaska Native, Asian/Pacific Islander (hereafter, Asian), Black/African American (hereafter, Black), Hispanic/Latinx (hereafter, Hispanic), non-Hispanic White, non-White, and White. Race/ethnicity was categorized as “Other” if not defined in the manuscript (i.e. labeled as “Other” or “Multi-racial” with no further definition) or could not be assigned to one of the above race/ethnicity groups. Study interventions were subdivided by type into “treatment” (i.e. surgery, chemotherapy, endocrine therapy, or radiation therapy), “psychosocial” (i.e. patient education, outreach, quality of life etc.), “alternative therapy” (i.e. yoga, meditation, vitamin supplementation, etc.), and “other” (i.e. genetics, risk stratification/prognostics, imaging/screening, etc.). Funding source was categorized into government-sponsored, industry-sponsored, and private funding. If more than one funding source type was listed, all sources were tallied. Social determinants of health were recognized as any of the following: income, education level, employment, housing, language, and insurance status.

To account for the varying proportional distribution of race/ethnicity groups comprising the USA population and the differing rates at which breast cancer affects those same groups, Participation to Prevalence Ratios (PPRs)^9^ were calculated for each trial to measure how reflective the trial population was of the general population with breast cancer. PPRs were determined by dividing the percentage of subjects with breast cancer in the clinical trial who were of a particular race/ethnicity by the percentage of the general population with breast cancer that are of that race/ethnicity, with a PPR less than 0.8 indicating that persons of X race/ethnicity group were underrepresented in a given clinical trial and a value over 1.2 signifying overrepresentation. Since participants being enrolled in breast cancer clinical trials generally have active disease and are typically diagnosed within a year of enrollment, we utilized incidence (i.e. rates of diagnosis of breast cancer) among each race/ethnicity group as the denominator that would more closely estimate the prevalence of “active” breast cancer instead of overall prevalence, which would include patients potentially living many years in remission with a history of breast cancer. The PPR denominator for each race/ethnicity group among the population with breast cancer in the USA was obtained from an analysis by Ellington et al., of trends in breast cancer incidence by race/ethnicity, which reported the following: Asian 3.5%, Black 10.5%, Hispanic 7.1%, and White 78.0%.^10^

The primary outcome measured was the rate of reporting the race or ethnicity of trial subjects. Secondary outcomes were the reporting of at least one subject from all four of the most prevalent race/ethnicity groups in the USA (Asian, Black, Hispanic, White), the use of race/ethnicity in the analysis of the trial, and the rate of equitable inclusion of racial minorities (measured as a PPR > 0.8 for Asian, Black, and Hispanic subjects concurrently).

Descriptive analyses were performed to determine the rate of reporting race/ethnicity by year during the study period. Bivariate analyses were performed to determine whether clinical trial characteristics were associated with higher rates of race/ethnicity reporting or inclusion of minority populations. For funding, studies may have had multiple sources, and therefore, categories were analyzed as individual factors. Infrequent intervention types (imaging/screening, genetics, prognosis/risk stratification) were grouped together for analysis as “Other.” Continuous data were compared using the Student’s *t*-test, and categorical data were compared using Pearson *χ*^2^ and Fisher’s exact tests as appropriate. For race/ethnicity reporting, multivariable regression was used to validate any variable with a *p*-value < 0.1 in the bivariate analysis. A *p*-value < 0.05 was considered statistically significant. SPSS, version 28 (IBM Corp, Armonk, New York), was used.

This study was deemed exempt from requiring Institutional Review Board approval since the data collected for this study was published in deidentified form and publicly available.

## Results

**Fig. 1:**
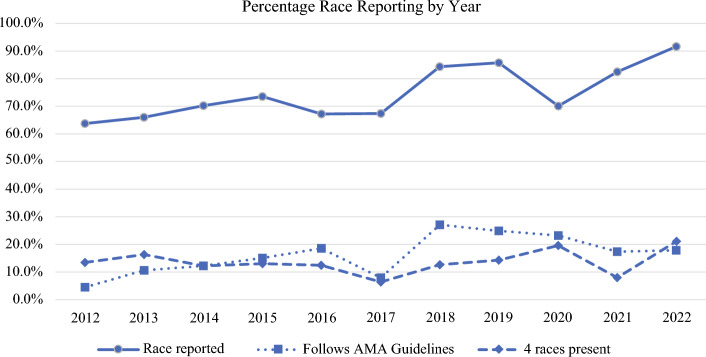
Degree of race reporting and relative inclusion of racial minorities in breast cancer clinical trials by year 2012–2022. AMA American Medical Association

From 2012 to 2022, 1965 human breast cancer clinical trials were published as a full manuscript in English. Among this group, 1068 trials (*n* = 871,683 subjects) met inclusion criteria and underwent further analysis. Race/ethnicity of research subjects were reported in 785 (73.5%) clinical trials (*n* = 736,160), with a positive trend of reporting noted from 2012 to 2016 (68.8%; range 63.8–73.6% per year) to 2017–2022 (79.3%; range 67.4–91.7% per year, *p* < 0.001). At least one subject from the four most prevalent race groups in the USA was represented in 194 (24.7%) trials that reported race, with a similar increasing trend from 2012–2016 to 2017–2022 (19.8% to 30.0%, *p* < 0.001). Trends of race/ethnicity reporting rates annually are displayed in Fig. 1. Race/ethnicity was reported in accordance with AMA guidelines in 130 (16.6% overall; range 4.5%–27.1% per year) trials. Race/ethnicity was included as a factor in the outcome analysis in 68 (8.7%) of the 785 trials where race/ethnicity was reported.

**Fig. 2:**
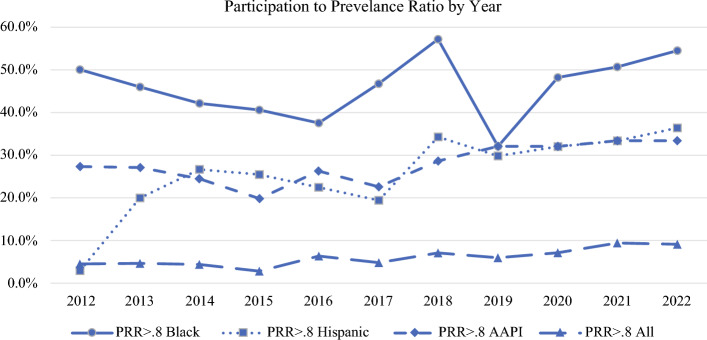
Proportion of overall subjects by race/ethnicity included in breast cancer clinical trials by year 2012–2022. PPR Participant to Prevalence Ratio, AAPI Asian American Pacific Islander

Of the 732,969 subjects in trials reporting race, 1692 (0.2%) were American Indian/Alaska Native, 16,551 (2.3%) were Asian, 43,288 (5.9%) were Black, 22,464 (3.1%) were Hispanic, and 570,897 (77.9%) were White. “Other” consisted of 77,562 (8.9%) subjects. Among the 785 trials that reported race/ethnicity, a Participant to Prevalence Ratio above 0.8 was achieved in 214 (27.3%) clinical trials for Asian subjects, in 351 (44.7%) for Black subjects, and in 215 (27.4%) for Hispanic subjects. A PPR > 0.8 for all three minority groups (Asian, Black, and Hispanic) concurrently was attained in 45 (5.7%) trials. Trends of race/ethnicity reporting and PPR annually are demonstrated in Figs. 1 and 2, respectively.

Clinical trial characteristics are presented in Table 1 according to reporting of race/ethnicity and inclusion of minority race groups. Trials were focused on traditional cancer treatment in 467 (43.7%), alternative therapies in 251 (23.5%), and psychosocial in 210 (19.7%). Trials were predominantly phase I (124; 11.6%), phase II (229; 21.4%), or “other” (549; 51.4%). The funding source was a government agency in 825 (77.2%), industry in 222 (20.8%), and a private entity in 456 (42.7%). Other SDH were collected in addition to race/ethnicity in 378 (35.4%) trials, of which 353 reported education level, 153 reported income, 77 reported insurance status, and 38 reported spoken languages.

**Table 1:** Characteristics of breast cancer clinical trials according to reporting of race/ethnicity and inclusion of minority subjects

Trial Characteristic	Total Clinical Trials *n* = 1068*	Race/Ethnicity Reported *n* = 785 (73.5%)	*p*-value	4 Race Groups Included *n* = 194 (24.7%)**	*p*-value	Race/Ethnicity Analyzed *n* = 68 (8.7%)**	*p*-value
*Publication Year*			< .001		< .001		.002
2012–2016	589 (55.1%)	405 (68.8%)		80 (19.8%)		47 (11.6%)	
2017–2022	479 (44.9%)	380 (79.3%)		114 (30.0%)		21 (5.5%)	
*Intervention Type*			< .001		.886		< .001
Treatment	467 (43.7%)	278 (59.5%)		68 (24.5%)		12 (4.3%)	
Alternative	251 (23.5%)	224 (89.2%)		52 (23.2%)		11 (4.9%)	
Psychosocial	210 (19.7%)	196 (93.3%)		52 (26.5%)		29 (14.8%)	
Other	140 (13.1%)	87 (62.1%)		22 (25.3%)		16 (18.4%)	
*Trial Type*			< .001		.666		< .001
Pilot	74 (6.9%)	60 (81.1%)		17 (28.3%)		3 (5.0%)	
Phase I	124 (11.6%)	62 (50.0%)		14 (22.6%)		0	
Phase II	229 (21.4%)	161 (70.3%)		43 (26.7%)		4 (2.5%)	
Phase III	89 (8.3%)	61 (68.5%)		11 (18.0%)		5 (8.2%)	
Phase IV	3 (0.2%)	0		0		0	
Other	549 (51.4%)	441 (80.3%)		109 (24.7%)		56 (12.7%)	
*Randomized Trial*			< .001		.524		.141
Yes	593 (55.5%)	503 (84.8%)		128 (25.4%)		38 (7.6%)	
No	475 (44.5%)	282 (59.4%)		66 (23.4%)		30 (10.6%)	
*Funding Source*							
Government			.029		.944		.233
Yes	825 (77.2%)	619 (75.0%)		153 (24.7%)		58 (9.4%)	
No	213 (22.8%)	144 (67.6%)		36 (25.0%)		9 (6.3%)	
Industry			< .001		.650		.056
Yes	222 (20.8%)	133 (59.9%)		35 (26.3%)		6 (4.5%)	
No	816 (79.2%)	630 (77.2%)		154 (24.4%)		61 (9.7%)	
Private			.651		.189		.766
Yes	456 (42.7%)	332 (72.8%)		90 (27.1%)		28 (8.4%)	
No	582 (57.3%)	431 (74.1%)		99 (23.0%)		39 (9.0%)	
None	30 (2.8%)	22 (73.3%)	.983	5 (22.7%)	.827	1 (4.5%)	.486
*SDH Collected*			< .001		.265		.515
Yes	378 (35.4%)	363 (96.0%)		83 (22.9%)		34 (9.4%)	
No	690 (64.6%)	422 (61.2%)		111 (26.3%)		34 (8.1%)	

SDH Social Determinants of Health

*Percentiles are calculated within the column among total clinical trials (*n* = 1068)

**Percentiles are calculated within each row among clinical trials reporting race (*n* = 785)

On bivariate analysis, higher rates of race/ethnicity reporting were significantly associated with later year of publication, psychosocial interventions, phase II studies, randomized trials, government funding sources, and the collection of other SDH. See Table 1. Number of enrolled subjects was not significantly different between studies reporting race/ethnicity (mean 933.7 ± 6,691.2 subjects) and those not reporting race/ethnicity (mean 490.2 ± 2,481.8 subjects, *p* = .277). Multivariable analysis demonstrated four independently correlated factors: year of publication “early” [Odd’s Ratio (OR) 0.521, 95% Confidence Interval (CI) .373–.730, *p* < 0.001]; phase II trial type compared with phase I (OR 1.789, 95%CI 1.108–2.890, *p* = 0.017); randomized trial design (OR 1.994, 95%CI 1.330–2.991, *p* < 0.001); and inclusion of other SDH (OR 8.351, 95%CI 4.484–15.500, *p* < 0.001).

Studies including race/ethnicity in the outcome analysis were significantly less likely to be published in the later cohort between 2017 and 2022 (5.5% versus 11.6%, *p* = 0.002), to be a treatment or alternative intervention type (4.3% and 4.9% versus 14.8% psychosocial and 18.4% Other, *p* < 0.001), and to be a phase I or II clinical trial (0 and 2.5% versus 8.2% phase III and 12.7% Other, *p* < 0.001). Table 1. The highest rates of studies achieving a PPR > 0.8 for all three minority race groups (Asian, Black, and Hispanic) concurrently were those published in the later cohort (2017–2022), of a psychosocial intervention type, with a nonrandomized trial design, or of an industry-sponsored or private funding source. Of these, only private funding was significant (9.0% versus 3.5%, *p* = 0.001), while all others did not have significant associations. Table 2.

**Table 2:** Rates among breast cancer clinical trials reporting race that achieved Participation to Prevalence Ratio (PPR) >0.8 for minority race/ethnic groups

Trial Characteristic *n* = 785	PPR >0.8 Asian *n*=214 (27.3%)	*p*-value	PPR >0.8 Black n=351 (44.7%)	*p*-value	PPR >0.8 Hispanic *n*=215 (27.4%)	*p*-value	PPR >0.8 All Minority *n*=45 (5.7%)	*p*-value
*Publication Year*		.067		.192		.056		.109
2012–2016 (*n* = 405)	99 (24.4%)		172 (42.5%)		99 (24.4%)		18 (4.4%)	
2017–2022 (*n* = 380)	115 (30.3%)		179 (47.1%)		116 (30.5%)		27 (7.1%)	
*Intervention Type*		< .001		.508		.010		.514
Treatment (*n* = 278)	100 (36.0%)		130 (46.8%)		60 (21.6%)		15 (5.4%)	
Alternative (*n* = 224)	40 (17.9%)		93 (41.5%)		71 (31.7%)		12 (5.4%)	
Psychosocial (*n* = 196)	48 (24.5%)		85 (43.4%)		65 (33.2%)		15 (7.7%)	
Other (*n* = 87)	26 (29.9%)		43 (49.4%)		19 (21.8%)		3 (3.4%)	
*Trial Type*		< .001		.573		.003		.506
Pilot (*n* = 60)	21 (35.0%)		24 (40.0%)		16 (26.7%)		3 (5.0%)	
Phase I (*n* = 62)	26 (41.9%)		28 (45.2%)		16 (25.8%)		2 (3.2%)	
Phase II (*n* = 161)	57 (35.4%)		80 (49.7%)		36 (22.4%)		10 (6.2%)	
Phase III (*n* = 61)	18 (29.5%)		24 (39.3%)		6 (9.8%)		1 (1.6%)	
Phase IV (*n* = 0)	0		0		0		0	
Other (*n* = 441)	92 (20.9%)		195 (44.2%)		141 (32.0%)		29 (6.6%)	
*Randomized Trial*		.007		.037		.480		.557
Yes (*n* = 503)	121 (24.1%)		211 (41.9%)		142 (28.2%)		27 (5.4%)	
No (*n* = 282)	93 (33.0%)		140 (49.6%)		73 (25.9%)		18 (6.4%)	
*Funding Source*		< .001		.919		.201		.554
Government								
Yes (*n* = 619)	145 (23.4%)		278 (44.9%)		179 (28.9%)		35 (5.7%)	
No (*n* = 144)	61 (42.4%)		64 (44.4%)		34 (23.6%)		10 (6.9%)	
Industry		< .001		.790		.031		
Yes (*n* = 133)	65 (48.9%)		61 (45.9%)		27 (20.3%)		6 (13.3%)	.455
No (*n* = 630)	141 (22.4%)		281 (44.6%)		186 (29.5%)		39 (6.2%)	
Private		.226		.291		.129		
Yes (*n* = 332)	97 (29.2%)		156 (47.0%)		102 (30.7%)		30 (9.0%)	.001
No (*n* = 431)	109 (25.3%)		186 (43.2%)		111 (25.8%)		15 (3.5%)	
None (*n* = 22)	8 (36.4%)	.331	9 (40.9%)	.716	2 (9.1%)	.051	0	.241
*SDH Collected*		< .001		.055		.224		.387
Yes (*n* = 363)	73 (20.1%)		149 (41.0%)		107 (29.5%)		18 (5.0%)	
No (*n* = 422)	141 (33.4%)		202 (47.9%)		108 (25.6%)		27 (6.4%)	

Percentiles are calculated within each row among clinical trials reporting race (*n* = 785)

PPR Participation to Prevalence Ratio, SDH Social Determinants of Health

## Discussion

In this study of 1068 USA breast cancer clinical trials published between 2012 and 2022, overall race/ethnicity reporting was 74%, with an increasing trend over time to as high as 92% of studies published in 2022. However, inclusion of minority populations in breast cancer clinical trials was critically low, with only 25% of race/ethnicity-reporting studies including subjects from all four primary race/ethnicity groups in the USA (Asian, Black, Hispanic White, Non-Hispanic White) and just 6% of trials having PPRs greater than 0.8 for Asian, Black, and Hispanic subjects concurrently. Furthermore, meaningful inclusion of race/ethnicity by way of analyzing trial outcome according to race/ethnicity occurred in only 9% of trials. These results indicate that while reporting of race/ethnicity has improved significantly over the past decade, the quality of reporting and the overall inclusion of minorities in breast cancer clinical trials require further attention and optimization.

Distributive justice, one of three core ethical principles of clinical research outlined in the Belmont Report, dictates that the burdens and benefits of research should be equitably distributed among research participants.^11^ Underrepresentation of minority race/ethnicity groups in clinical research signifies a failure to meet this ethical standard as these populations may not benefit from the research being conducted. Fisher et al. noted that Black and Hispanic enrollment in phase I clinical trials evaluating safety and adverse events is higher in the northeastern (42%) and southwestern (55%) USA at rates more than double their representation in the USA population.^12^ This, coupled with very low rates of inclusion in later-phase and other randomized clinical trials, is worrisome as racial/ethnic minorities may be unfairly bearing the burden of safety research at higher rates, while not being afforded equitable opportunities in trials where they may derive benefit. Furthermore, clinical trials underpowered to detect racial/ethnic differences in outcomes may contribute to the persistence of health disparities.

This study demonstrated the highest rates of race/ethnicity reporting in trials published in more recent years, with a randomized trial design, phase II trials compared to phase I, and those collecting SDH. The greatest opportunities for improvement, however, remain clinical trials investigating traditional cancer treatments and those that are industry-sponsored, both demonstrating race/ethnicity reporting rates under 60% among the trials in our cohort. Our analysis also noted deficiencies in the quality and equity of reporting race and ethnicity as outlined in best-practice guidelines published by the AMA.^7^ These guidelines dictate that specific race categories are used instead of the terms “persons of color” or “non-White,” race groups are capitalized and alphabetized, race is used as an adjective not a noun, and that the race/ethnicity identification method is described. In this study, AMA guidelines were adhered to in just 4.5% of breast cancer clinical trials published in 2012, reaching only 27.1% of trials in 2018, and ultimately falling to just 18.0% in 2022. Since the AMA guidelines were only recently published in 2021, these results present more of a baseline to measure compliance going forward. We did note that the specificity of race/ethnicity reporting, while not up to the standards of the AMA, did appear to have marginally improved over the study interval. Subjects defined as ‘Non-White’ comprised 15–20% of study subjects 2017–2019 while rates for those designated as Black or Hispanic registered near zero. Then, in 2021, the rates of Black and Hispanic subjects each increased to 15–20% of studies while the proportion of ‘Non-White’ subjects plummeted to near zero, seemingly signifying that more recently published trials utilized more specific descriptions of their “Non-White” study participants.

While manuscript reporting of the race/ethnicity of subjects improved over time, this study aimed also to evaluate for a concomitant increase in inclusion of minority race/ethnicity groups in the conduct of breast cancer clinical trials. Inclusion was measured in several ways (overall proportion of racial/ethnic groups among total subjects, rate of inclusion over time, enrollment of at least one member of each race/ethnicity group, and proportionally adjusted rates of inclusion using PPR) and was noted to be critically low in each of these metrics in the present study. When looking at the overall proportion of inclusion of subjects by race/ethnicity, Asian participants comprised 2% of trial subjects, with Black and Hispanic participants 6% and 3%, respectively. Similar rates were reported by Loree et al. in their study of race/ethnicity representation in cancer drug approval trials 2008 to 2018, with Black race reported in 3.1% of subjects and Hispanic ethnicity in 6.1%.^13^ Unger et al. also found that Black patients were underrepresented in industry-sponsored trials (2.9%) compared with government-sponsored trials (9.0%) in cancer drug approvals 2008–2018.^14^ This is a disheartening finding given that Black Americans are diagnosed with significantly higher rates of triple-negative breast cancer (19% in Black patients compared to 9% for White patients)^15^ and novel chemotherapy and immunotherapy interventions, which are largely investigated via industry-sponsored trials, present a critically important avenue toward improvement in triple-negative breast cancer outcomes. Underrepresentation of Black subjects in these clinical trials limits applicability of findings to the patient group that may significantly benefit. Evaluating inclusion over time, overall proportions of Asian, Black, and Hispanic study participants did not increase in our study, with rates in 2022 similar to those in 2012. In a more limited study of 98 breast cancer clinical trials identified in ClinicalTrials.gov, Keegan et al. likewise found that overall representation of non-White enrollees did not increase from 2010 to 2022.^16^ However, we did find that the proportion of studies reporting at least one member from all four of the most prevalent race groups in the USA improved to over 50% in 2022 from under 20% in 2012.

Though important to track overall proportions of inclusion of racial minorities in studies, Asian individuals constitute only 6.4% of the American population according to the USA 2020 Census, with Black persons comprising 13.7% and Hispanic persons 19.5%.^17^ Measures of inclusion must, therefore, account for this relative proportional difference in prevalence of minority groups in the USA population. Furthermore, rates of breast cancer vary among different race/ethnicity groups, with White women diagnosed at a rate of 133.7 per 100,000 compared with 127.8 in Black women and 99.2 in Hispanic women.^14^ Participant to Prevalence Ratios provide a mechanism by which to measure the inclusion of individuals of a particular race group diagnosed with breast cancer among breast cancer study subjects overall, with a PPR greater than 0.8 indicating adequate proportional representation of a specific race group. In the current study, achieving a PPR > 0.8 for Black subjects remained relatively steady from 2012 to 2022 at around 50% of trials, while a PPR > 0.8 for Hispanic subjects increased slightly from 20% of trials in 2013 to 35% in 2022. Achieving a PPR > 0.8 for Asian subjects also had a slight increase from 27.1% in 2012 to 33.3% in 2022. Ideally, a clinical trial achieves a PPR > 0.8 for all three minority groups to ensure that trial results are generalizable to all minority groups in the USA population. While an overall PPR > 0.8 for all three minority subjects optimistically doubled from 4.7% of studies published in 2012 to 9.1% in those from 2022, this remains a gross underrepresentation of minority groups overall, with lowest rates observed among trials investigating traditional treatment modalities, government-sponsored trials, and randomized trials. This indicates that even when controlled for varying prevalence of race/ethnic groups in the USA, inclusion of minority race/ethnicity groups remains critically low, and efforts should be made to increase diversity in breast cancer clinical trial research.

Several barriers to the inclusion of minority race groups in clinical trials have been enumerated and include, but are not limited to, the distrust of subjects toward researchers after horribly unsound research methodology led to the unchecked harm of Black patients in Tuskegee, inaccessibility due to transportation issues, inability to miss work/childcare responsibilities, lack of opportunity owing to unawareness of trials or trials not open to enrollment nearby, noninclusion due to implicit biases of researchers assuming ineligibility or disinterest in participation, or inability to participate owing to comorbidities, substance use, or language and communication issues.^18,19^ To address these barriers, a multi-faceted approach is necessary. Clinical trials should attempt to include enrollment sites located in high-diversity states. USA Census data show that more than 30% of the populations of California, New Mexico, Puerto Rico, Arizona, and Texas is Hispanic, and more than 30% of the populations of the District of Columbia, Louisiana, and Mississippi is Black. The Asian population comprises more than 30% of Hawaii, 15% of Californians, 9% of Washingtonians, and 7% of persons in Massachussetts.^20^ Trials should also consider partnering with community groups to outreach to specific racial/ethnic groups.^19^ Producing outreach materials with images of non-White skin colors, translated into non-English languages, increased readability at lower reading levels, and consideration of virtual/electronic methods to follow participants rather than in-person visits is key.^21^ An industry now exists to help enhance clinical trial diversity, with companies such as PhRMA and Antidote available to connect researchers with underrepresented patients.^21,22^ In June 2024, the U.S. Food and Drug Administration also published an online draft of its plan to require study sponsors to formulate Diversity Action Plans that outline methods to determine diversity quotas and strategies to enroll diverse study populations.^23^

The limitations of this study are that race/ethnicity definitions were inconsistent across the clinical trials included in this analysis. American Indian/Alaska Native subjects were not specifically evaluated for PPR given that the group comprises just 1% of the USA population and only 0.2% of our study population. We were also not able to isolate the demographics of subjects who were approached to participate in the clinical trials but were not ultimately enrolled, which may have altered our findings on inclusion and further inform efforts to combat barriers to inclusion in clinical trials. In addition, the measures utilized in this study for inclusion are likely overestimations of the true rates of inclusion of minority race groups given that we included only the studies where race was reported (*n* = 785) as the denominator instead of all studies published (*n* = 1068) as we could not assume that studies not reporting race did not include subjects from Non-White groups. It is possible, however, that trials reporting race included more Non-White subjects than those not reporting race, which could erroneously elevate proportions reported in this study. Furthermore, the PPRs calculated in this study utilized rates published in 2018, which may not have been reflective of race/ethnicity distributions throughout the entire study period. For instance, the 2020 Census reported that the Hispanic population grew by 23% from 2010 to 2020.^17^ In addition, the PPR denominators utilized in this study reflected race/ethnicity distributions for new breast cancer diagnoses overall which may not represent the specific demographic of breast cancer subpopulations in a given trial such as triple-negative breast cancer (with a higher rate of Black patients diagnosed) or stage IV breast cancer (where patients can live many years after diagnosis). Despite these limitations, our study is one of the largest evaluations of racial/ethnic representation across more than 1000 breast cancer clinical trials and over 800,000 participants.

## Conclusions

Although race/ethnicity reporting rates significantly improved over time, minority populations remain gravely underrepresented in breast cancer clinical trials, limiting the generalizability of trial results in these populations, and possibly contributing to the widening of gaps in health disparities. Efforts focused on addressing barriers to participation and improving recruitment strategies to meaningfully include minority subjects in clinical trials are vitally important to improving cancer care for underrepresented populations.
